# Following Up Crack Users after Hospital Discharge Using Record Linkage Methodology: An Alternative to Find Hidden Populations

**DOI:** 10.1155/2015/973857

**Published:** 2015-09-06

**Authors:** Veralice Maria Gonçalves, Rosemeri Pedroso, Antônio Marcos dos Santos, Lisia Von Diemen, Flavio Pechansky

**Affiliations:** ^1^Center for Drug and Alcohol Research, Clinics Hospital of Porto Alegre, Federal University of Rio Grande do Sul, 400 Professor Alvaro Alvim Street, 90420-020 Porto Alegre, RS, Brazil; ^2^Information Technology Department of the Unified Health System, National Ministry of Health, Brazil

## Abstract

This paper presents the probabilistic record linkage (PRL) methodology as an alternative way to find or follow up hard-to-reach population as crack users. PRL was based on secondary data from public health information systems and the strategy used from standardization; phonetic encoding and the rounds of matching data were described. A total of 293 patient records from medical database and two administrative datasets obtained from Ministry of Health Information Systems were used. Patient information from the medical database was the identifiers to the administrative datasets containing data on outpatient treatment and hospital admissions. 40% of patient records were found in the hospital database and 12% were found in the outpatient database; 95% of the patients were hospitalized up to 5 times, and only 10 out of them had outpatient information. The record linkage methodology by linking government databases may help to address research questions about the path of patients in the care network without spending time and financial resources with primary data collection.

## 1. Introduction

In 2012, about 3.5 to 7.0 percent of the world population aged between 15 and 64 had used an illegal drug at least once in a previous year [1]. In the Americas, the most prevalent primary drug of abuse among those seeking treatment was cocaine [1]. In Brazil, crack-cocaine consumption has become more prominent; however, treatment of this specific type of drug use remains a challenge [2, 3]. Longitudinal follow-up studies about this type of drug use often cannot find these patients at the appropriate level of retention and there are too many losses, since they seem to evade after hospital discharge. Attempts to follow up this hidden and hard-to-reach population for evaluating the effectiveness of interventions face methodological difficulties. Furthermore, longitudinal studies and clinical trials are conducted at very high costs. One option to overcome these limitations is to utilize another method for evaluating interventions: the record linkage methodology based on secondary data available from the existing information systems.

Record linkage techniques based on medical and administrative databases have been used on epidemiological studies in several countries. These studies generally involve the use of hospital, population, and outpatient data, which allow for the collection of comprehensive patient information and for more robust analyses [4, 5]. Retrospective cohort studies have been employed in Canada [4], Australia [6], and the United States [7], as well as in a number of European and Scandinavian countries [8], to investigate issues such as cause-specific mortality [9], hospital admissions [10], prevalence of mental disorders [11], and antecedents of deliberate self-harm [12].

Record linkage (RL) studies involve the comparison of data in medical and administrative databases with the aim of finding records that are believed to relate to the same patient. Cases in which two or more records relating to the same individual are identified and linked are referred to as exact matches and are especially frequent when the databases involve similar patient numbering systems. Conversely, exact matches are far less likely when databases do not assign patients with exclusive identifiers, when data entry is not standardized, or when files are too large [13].

In Brazil, digital public health databases created from the national health information system are used for obtaining data for a wide variety of purposes. Some databases are maintained for administrative reasons, while others are used for recording and reporting epidemiological data. These databases contain a large number of records; however, since data entry is not standardized, it is challenging to link records relating to the same patient across multiple databases, which limits the practical application of RL in this setting.

Despite a growing trend in the use of medical records in Brazilian research [14–16], there is scarce information on how applicable the use of recorded data to follow up psychiatric patients is, particularly in the case of individuals with substance use disorders.

The present study describes the use of record linkage to a cohort of drug users in both inpatient and outpatient treatment during a period of three years. We also present this method as an exercise for further use in studies focused on patients follow-up and on either retrospective or prospective analysis of their paths throughout the care network and the continuity of care.

## 2. Methods

Three databases were used in this study: (1) a database containing information about 293 crack users admitted for inpatient treatment in two psychiatric institutions between January 2010 and December 2012 (primary data considered the main database); (2) an administrative secondary database containing public hospital admissions records; (3) an administrative secondary database containing public outpatient treatment. The second dataset was obtained from the Ministry of Health (MoH) reports on public hospital admissions, and the third was obtained from the outpatient treatment registry of the Brazilian MoH.

The study sample comprises both patients hospitalized and patients treated in outpatient clinics of the state of Rio Grande do Sul, Brazil, who received diagnoses in the F00-F99 (Mental and Behavioral Disorders) code group of the ICD-10 (International Code of Diseases, 10th review) between January 2010 and December 2012.

A sample of 293 discharged crack users recorded in the first database was linked to the Hospital Admissions Database and to the Outpatient Treatment Database.

The Hospital Admissions Database contained information on all patients hospitalized in public health services during the period of the study. The database was compiled from patient discharge data provided by public hospitals to the MoH. This dataset contained information on variables such as hospital and patient identification, hospitalization characteristics, patient diagnosis, and procedures conducted in a sample of 604,877 individuals.

The third database was constructed similarly to the second, using outpatient specialized treatment data provided by public health facilities to the MoH. This database contains information such as facility characteristics, patient diagnosis, procedures conducted, and patient identification. In this database, patient identification data were recorded for patients undergoing specialized procedures, such as attending psychosocial care centers. A sample of 495,902 records was obtained from this dataset.

Both information systems (hospital and outpatient) were required by law to make use of a particular patient identifier (a National Health Card number). Although this is a nationwide system, its implementation is still underway and is occurring with variable efficiency in different Brazilian states. Because of the different stages of implementation, this identifier could not be used in this study. For security and ethical reasons, patient identifiers and other personal information are not available for public access. Therefore, access to these databases in the present study was only possible after a formal request to the MoH and approval from an Institutional Review Board. This approval was granted based on resolutions passed by the Brazilian National Health Council. Personal information was available in the databases, but no individual identifier was analyzed.

Patient first and last names, gender, and date of birth were used for determining exact matches, while first and last names as well as gender were used for identifying possible matches. A phonetic encoding function was used for linking records between the databases.

Data linkage, especially between databases with a large number of records, is facilitated when each record in the dataset is unambiguously identified by a single numeric identifier such as a social insurance number. In computerized databases such as patient registries, there may be the need to retrieve nonnumeric data, such as patient names. This may be particularly important for confirming exact matches or for excluding duplicate reports of the same person. However, the search for patients by name can be challenging, especially in the case of names with the same pronunciation but divergent spellings. Examples of such names in Brazilian Portuguese include Luiz and Luis, Cristiane and Christiane, Fernandes and Fernandez, and Gonsalves and Gonçalves. One way to facilitate data searching by patient name is through phonetic encoding. This technique can increase the likelihood of identifying records belonging to the same patient across different databases. Phonetic encoding involves the transformation of written text into phonological words. This procedure can be used to identify the equivalence between words or word combinations which differ in spelling but are phonetically similar, since the phonetic output of equal sounding words is the same, regardless of spelling differences. The first step in this process is the standardization of fields for phonetic encoding. This step required the standardization of data fields across databases, such as sex and birth date. The same coding system was implemented for these variables in all databases used (1 for male, 2 for female; ddmmyyyy format for birth date). After standardization, all accents and special characters were removed from data strings.

The phonetic encoding of patient names was then performed using a code developed by the present authors which is similar to the Soundex Code [17], but specific to the Portuguese language.

The goal of record linkage is to bring together information from two or more records believed to relate to the same unit, which can consist of a person, a family, or even an institution. These records may exist in duplicate in the same file or consist of distinct records in more than one database. The result of the identification and linkage of records related to the same unit across databases is called an exact match.

Record linkage strategies can be classified as either deterministic or probabilistic. Deterministic record linkage consists of the identification of multiple records which agree exactly with designated components, referred to as match keys. In probabilistic record linkage, pairs of records are classified as links, possible links, or nonlinks and can be used when entries do not have exclusive numerical identifiers. The present study used probabilistic record linkage to obtain statistical data for researching purposes. The results of our record linkage process were used for discovering and analyzing relationships between variables which would allow us to identify the individual characteristics and patient outcomes in the state of Rio Grande do Sul, Brazil.

*The Record Linkage Process*. The process was performed in all three databases through the following steps:

1. Similar fields were standardized (sex and date of birth).
2. All accents and special characters were removed from patient names.
3. Patient names were phonetically encoded using an algorithm specifically designed for the Portuguese language.
4. The main database (Crack User Hospital Admission Dataset) was used to perform a first record linkage run by searching the fields First Name + Last Name + Sex + Date of Birth in the Inpatient Database.
5. Matching records were recorded in the Exact Links Database and in the Inpatient Matches Database.
6. The nonmatching records returned in step (4) were used for a second record linkage run, by searching the fields First Name + Last Name + Sex.
7. Matching records were entered into a Possible Links Database.
8. Nonmatching records remaining after step (6) were entered into a Nonlinks Database.
9. Matches in the Possible Links Database and the Crack User Hospital Admission Form were confirmed by contacting patients to inquire about the hospitalization data.
10. Matches resulting from the procedures in step (9) were recorded in the Inpatient Matches Database, together with the previously established exact links.
11. Remaining records were entered into the Nonlinks Database.

After the linkage process, the final file with the retrospective and prospective path of the crack users cohort was exported to XLS format and then analyzed with PASW, 18th version.

**Figure 1:**
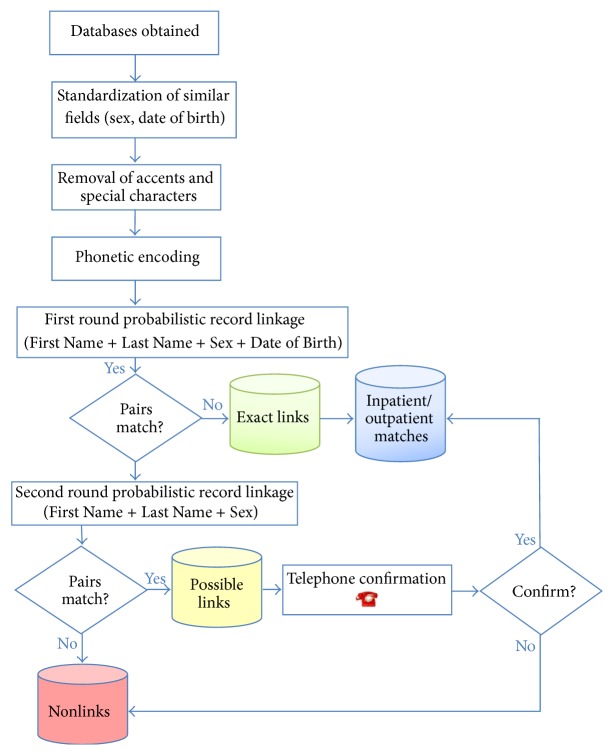
Flow chart of record linkage steps.

The record linkage process used in this study is presented in Figure 1.

## 3. Results and Discussion

**Figure 2:**
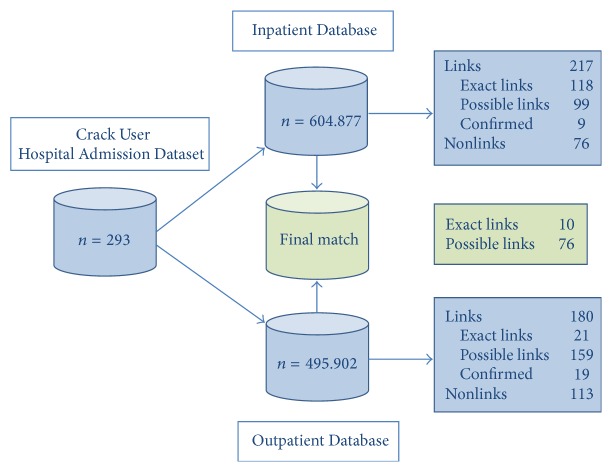
Record linkage matching process.

Record matches retrieved from the three databases are presented in Figure 2.

Thirty percent out of the 293 patients were teenagers (aged 12–18). The sample was predominantly male (94%). The mean age of the adolescents was 15.57, and among adults the mean age was 30. All patients were hospitalized for crack-cocaine treatment in two institutions, corresponding to a homogeneous sample.

A total of 217 patients out of the 293 records from the main database were located in the hospital database, corresponding to 74% of the total. Among these, 118 (54%) patients were considered exact links or exact matches due to the similarity of first and last surnames, sex, and date of birth. The other 99 (46%) were considered possible links, as their records did not match in the initial search, but paired in a subsequent search using the match key – surname + first name + sex. The 76 remaining records analyzed, approximately 25% of the total, were considered nonlinks.

After the completion of this step of linkage, a telephone survey was conducted to confirm the possible links found. The process consisted of calling up the contact numbers reported by patients at admission to find them and confirm the hospitalization data. Only 9 out of the 99 possible links were confirmed.

One hundred and eighty links (61% out of the 293 patients) were established between comprised were exact matches, whereas 159 (88%) were possible links. The remaining 113 (39%) records were considered nonlinks. After this step of linkage, we repeated the telephone survey process, then obtaining 19 treatment confirmations as exact matches.

Analysis of the two linked databases (obtained from comparing the main dataset with hospital admissions dataset and comparing the main one with outpatient treatment dataset) revealed only 10 exact links and 76 possible links. This last step linked the 127 patients (hospital dataset) with the 40 patients (outpatient dataset). The exact links obtained revealed that approximately 8% of the discharged patients had undergone outpatient care.

The study demonstrated that the record linkage methodology can be used to follow up hidden and hard-to-reach individuals; this could be related to treatment outcomes of drug users but could also be expanded to other samples. Based on this methodology, the use of federal data benefits researchers and state agencies and qualifies health data produced all over the country.

The main strength of this study was its large sample, which included the records of all patients discharged during the period of the study, regardless of their psychiatric or medical diagnosis. By linking government databases, it is possible to get valuable information without spending time and financial resources with primary data collection. Depending on the research purposes, the cost of obtaining a dataset from a sample with 1,000,000 patients, as the one we used in this study, is discouraging. Furthermore, systematic use of secondary databases strengthens existing data that are expensive, time consuming, and hard to obtain. This is one of the reasons to avoid further data collection of fragmented and costly data for individual studies and to strengthen the use of administrative government data. In particular, since the linkage rates for this study were not as high as expected, this can point out the need for improvement of quality in data recording. Thus, the information systems could be used at all for research purposes.

## 4. Conclusion

This study is important in demonstrating that probabilistic linkage of service use data can be valuable in tracking hard-to-reach populations in the Brazilian setting and may help address different types of research questions. Using available data in this way promotes better recording and addition of new or more useful data fields by data custodians who administer and supply the data. Although these data are primarily administrative tools for the agencies that own them, improvements in the aspects of data fields, collection methodology, and quality control procedures lead to better linkage rates and more confidence in research findings. Using these data for research purposes sheds light on the areas where these datasets can be improved for all users.

This type of study can be expanded to a nationwide scope including other health care databases, so that more comprehensive information could be obtained regarding both the management of public service networks and possible improvements to these services.
